# MDR-TB in Pakistan: Challenges, efforts, and recommendations

**DOI:** 10.1016/j.amsu.2022.104009

**Published:** 2022-06-17

**Authors:** Mahad Ahmed Khan, Wajeeha Bilal, Hanzla Asim, Zainab Syyeda Rahmat, Mohammad Yasir Essar, Shoaib Ahmad

**Affiliations:** a Dow University of Health Sciences, Pakistan; b Kabul University of Medical Sciences, Kabul, Afghanistan; c District Head Quarters Teaching Hospital, Faisalabad, Pakistan

**Keywords:** Tuberculosis, MDR-TB, COVID-19, Pakistan, MDR, multiple drug resistance, PMDT, Programmatic Management of Drug-Resistant Tuberculosis

## Abstract

Tuberculosis (TB), a global health concern is also a leading cause of mortality and morbidity across Pakistan affecting a major proportion of the population. The absence of an integrated system to control the spread of TB has led to a rise in multidrug resistant strains of TB (MDR-Tb) which do not exhibit any sensitivity towards the first line therapy for TB. Such adverse circumstances call for effective planning strategies to mitigate the health hazards of MDR-TB. This article briefly highlights the challenges encountered by the already burdened healthcare system and suggests relatively inexpensive approaches to tackle the ongoing crisis associated with MDR-TB on a national scale.

## 1 Introduction

Tuberculosis (TB) is an infectious bacterial disease caused by *Mycobacterium tuberculosis*, which is transmitted between humans by respiratory contact, most commonly affecting the lungs but can harm any tissue. For decades, TB has been a global health concern, despite significant medical and social interventions in developing countries. The burden of pulmonary tuberculosis in Pakistan is alarmingly high. The country is home to 210 million people, of which 1.5 million suffer from TB [1]. According to estimates, Pakistan ranks sixth in the world for the number of tuberculosis cases [1]. Since the advent of drugs, the emergence of strains with drug resistance and multiple drug resistance (MDR) has dramatically increased. This serious illness occurs when the bacterium is resistant to at least two of the most powerful first-line anti-TB drugs such as Rifampicin (RIF) and Isoniazid (INH) or at least resistant to four first-line drugs [2]. In the list of 30 high burden countries (HBC), Pakistan ranks fifth with an estimated 518 000 TB cases, including 15 000 MDR-TB cases [3]. The findings of various studies have led us to believe that young people are at a greater risk of contracting the disease [4]. It is due to poor dietary habits and nutrients that reduce immunity levels in the age group, particularly aged between 10 and 25 year [2]. Province wise, highest numbers of MDR cases were reported in Punjab (51%) which is followed by Sindh (23%), 15% in Khyber Pakhtunkhwa and 3.5% in Baluchistan [4]. Based on analyses of the increasing number of MDR-TB cases, it is found that most of the time they are associated with treatment failure, relapse, complications, and death [5]. Delay in diagnosis, inappropriate and inadequate drug regimens, poor follow-up, and lack of a social support program are key factors contributing to the emergence of drug resistance [4]. Based on an analysis and comparison of old and new data, a relatively high percentage of rifampicin drug resistance (4.2%) was found in newly diagnosed TB cases, followed by almost four times in previously treated cases [4]. Hence, Pakistan must adopt effective strategies for controlling the disease at an early stage and emphasize the necessity of intensifying TB awareness and management programs in conjunction with strict infection control measures. High-quality MDR-TB detection and awareness programs should be implemented, and treatments should follow WHO guidelines.

## 2 Challenges

The treatment of MDR-TB is challenging to provide around the world for various reasons including scarce resources and the production of adverse effects in treated patients [6]. A report from Iran found major adverse effects of MDR-TB treatment to be neurologic side effects, auditory toxicity, hepatitis, rash, and renal toxicity [7]. A major growing concern revolves around mutations in MDR-TB strains which have resulted in the creation of extensively-drug resistant TB (XDR-TB) [6]. XDR-TB strains are defined as MDR-TB strains that are resistant to second line injectable drugs as well as fluoroquinolones [8]. Worldwide, of the 558 000 MDR-TB cases registered in 2017, almost 8.5% of the cases were infected with XDR-TB [9].

In addition, many risk factors such as incorrect or inappropriate antibiotic intake and person-to-person transmission play a major role in the emergence of MDR-TB strains [6]. One study states that amongst the leading causes of infection with MDR-TB is previous history of a tuberculosis infection [10]. This finding provides a link between the importance of prevention of tuberculosis and the mismanagement in treatment; both predispose to an MDR-TB infection.

MDR-TB can also affect many areas of the body other than the lungs, such as the spine, brain, and kidneys [11]. Finding treatment options in the limited spectrum available to cure these superimposed dilemmas can prove increasingly difficult for healthcare professionals around the world, especially for those practicing in developing countries with extremely scarce resources. To add, patients with HIV have a high mortality rate if infected with MDR-TB or XDR-TB [12]. Some studies show a fatality rate of over 90% [12]. Another interesting correlation is that MDR-TB has been found to be almost twice as common in tuberculosis patients with HIV rather than tuberculosis patients without HIV [12].

The emergence of SARS-COV-2 has threatened to push the healthcare systems of many developing countries over the edge, and various effects of the pandemic have negatively affected the management of MDR-TB. For example, an article in India notes that disruptions in the supply chains of medications have resulted in an inability of some patients to obtain their necessary doses [13]. In addition, the mortality of patients infected with COVID-19 along with an underlying lung disease such as tuberculosis is high [14]. A recent study from Haiti states that patients infected with MDR-TB and COVID-19 are more prone to poor outcomes because of a noted decrease in lobar volume and an increased amount of cavities than people infected with drug-susceptible tuberculosis [14].

## 3 Efforts

The management of MDR-TB requires a robust and effective strategic plan at both the national and provincial levels. The government has adopted innovative approaches according to the WHO guidelines to deal with the multidrug resistant TB crisis in the country. In 2001, the declaration of TB as a national emergency instigated the Ministry of Health to create National TB Program (NTP) which by 2025, aims to reduce the incidence of TB by half as compared to the incidence rate in 2012. In 2009, WHO devised a TBIC (TB infection control) policy to prevent the spread of TB and this policy was adopted by Pakistan's national TBIC plan [15]. Employment of these policies led NTP to launch a Programmatic Management of Drug-Resistant Tuberculosis (PMDT) model of care in 2010 [16,17]. PMDT program of Pakistan consists of a macrosystem at the top which includes WHO and Global Fund and a microsystem at the bottom comprising of national and provincial TB programs and NGOs working together. Under this program, NTP established many clinics across the country providing free of cost consultation and medicines for drug resistant TB along with contact tracing under the mandatory TB case notification project (MCN). The initiation of these clinics led to the success rate reaching 76% as compared to 10%–46% previously and death rate declining from 40% to 13% by 2011 [17,18].

Along with NTP, provincial TB program (PTP) is also established in every province and has similar aims to eradicate TB by the coming decade. To shorten the gap between notified cases and actual cases, NTP established a Public-Private Mix model (PPM) in 2014 which had 4 categories of the public sector namely general practitioner (GP), TB care provided by NGOs, private hospitals, and other public sector (Parastatal) hospitals like WAPDA, Railway hospitals etc. PPM model led to increased case notifications in the age groups of <15 years and >54 years [19]. It also initiated Directly Observed Treatment, Short course (DOTS) in all the provinces according to WHO guidelines in which the patients are given short course drugs along with careful observation of treatment by the health care workers [20].

In a recent conference, namely End TB Summit held on January 18th, 2021, president, Dr Arif Alvi addressed the ongoing TB crisis by mentioning the number of TB cases detected annually estimated to be around 350 000 and emphasized on how finesse across the globe could be brought together to bringing together to help Pakistan put an end to TB by 2030 [21].

To minimize the impact on diagnosis and treatment of TB during COVID 19, several approaches were taken in different cities like Karachi which include co-screening for COVID 19 and TB in pre-existing TB centers, home delivery of medicines, maintenance of protocols for the safety of both the patients and healthcare workers, closely monitoring the mental well-being of health care workers and calling in to check up on the patients who test positive [22].(Table 1)

**Table 1:** Surveillance programs operating across the country.

Surveillance Programs	Functions
National Tb Program (NTP) [15]	It operates according to WHO guidelines and aims to reduce the incidence rate of TB and the number of deaths by Tb to nearly zero.
National TBIC (Tb infection control) plan [15]	Aims to minimize the risk of spread of TB among the health care workers by providing TBIC supplies such as masks, education, and training.
Programmatic Management of Drug-Resistant Tuberculosis (PMDT) [17]	Establishment of clinics across the country to ensure free provision of cost consultation and drugs for treatment along with some financial support for patients covering their transportation expenses
Mandatory Case Notification (MCN) project [18]	Its objective is to increase the number of reported cases so that timely diagnosis and treatment can be achieved along with contact tracing
Provincial TB Program (PTP) [19]	Works hand in hand with NTP to eradicate TB from Pakistan and provides a systematic approach on the provincial level in managing the risk of spread of TB
Public-Private Mix model (PPM) [19]	Established by NTP so that the public and private sector work together towards the goal of preventing the spread of TB along with better treatment strategies for TB

## 4 Recommendations

The most important thing that the public and private healthcare sectors can do to prevent the spread of MDR TB (multidrug resistant tuberculosis) is to raise awareness amongst the masses to take all the medications exactly as prescribed by the health care providers, none of the doses should be missed nor the treatment should be stopped earlier. This can be done through campaigning, posters, brochures, TV commercials etc. [23] DOTS refers to direct observed therapy which means that a trained healthcare worker or family member provides the prescribed drugs and watches patient swallow every dose. This strategy is critical for patients with drug resistant TB. TB is more common in large sized underprivileged families whose monthly income is less than average treatment cost of TB. The high cost of medications of TB along with the cost associated with travelling to the health centers daily for DOTS creates barriers in the treatment completion of TB patients. To combat this health organizations should set up camps in underprivileged TB hotspots areas where DOTS treatment should be administered free of cost. Such efforts will also prove to be effective in saving patient's time encouraging them to complete their treatment course.

Delay in the diagnosis of TB is a major contributor for the current transmission of TB in Pakistan so early diagnosis and treatment are crucial for effective MDR-TB control strategy. National Institute of Health should undertake the F-A-S-T strategy i.e., to Find Cases Actively by cough surveillance and rapid molecular Sputum testing, Separate safely, and Treat based on the rapid Drug Susceptibility Test (DST) [24]. The FAST strategy is built on a renewed appreciation of evidence showing that effective TB treatment reduces TB spread rapidly, even before sputum smear and culture turn negative. NIH has not yet implemented this method, but it has the potential to be a game changer if implemented fast.

Measures should be taken to make it obligatory for the infected patients in the hospitals to wear surgical masks as it reduces aerosol transmission to a great extent. Public and Private health care sectors should be provided free N-95 masks for the health care workers especially those working in the PMDT clinics as they are the most effective to stop transmission of airborne TB particles but are expensive. Apart from that, healthcare professionals should be instructed on how to properly wear these masks, which must be airtight and worn at all times while interacting with patients [24].

Lastly most importantly National Institute of Health should make it a priority in their National TB program strategy to screen close contacts of the patient which will help in early detection of new cases. This can be done by molecular typing. Molecular typing is a way of identifying specific strains of microorganisms by looking at their genetic material mainly used to pinpoint the source of outbreaks. Molecular typing and social network analysis can be effective for the health officials to identify high risk groups and community locations where MDR TB transmission is occurring [24].

TB and COVID-19 are both respiratory illnesses. They share a couple of same systems such as cough fever, shortness of breath, fatigue, and loss of appetite. Due to cofounding clinical symptoms of TB and COVID-19, routine screening of TB must be encouraged by NIH amongst confirmed cases of COVID-19 patients coming from areas which are TB hotspots.

## 5 Conclusion

The emergence of MDR-TB has made the management of TB extremely challenging. The prevalence of such resistant strains of TB amongst the Pakistani population is alarmingly high due to inadequate awareness sessions on the transmission of this deadly respiratory infection, non-adherence of infected individuals to the drug regimens and lack of preventive measures practiced by the general population. The progress so far, to eradicate MDR-TB has been sluggish and insufficient. Hence, strict measures by the public and private healthcare sectors should be introduced on a wide scale to ensure maximal reduction of MDR-TB cases.

## Please state any sources of funding for your research

None.

## Ethical approval

None.

## Consent

N/A.

## Author contribution

All authors equally contributed.

## Registration of research studies

1.Name of the registry:
2.Unique Identifying number or registration ID:
3.Hyperlink to your specific registration (must be publicly accessible and will be checked):

## Guarantor

N/A.

## Funding

None.

## Declaration of competing interest

None.
